# Association of marital/partner status with hospital readmission among young adults with acute myocardial infarction

**DOI:** 10.1371/journal.pone.0287949

**Published:** 2024-01-26

**Authors:** Cenjing Zhu, Rachel P. Dreyer, Fan Li, Erica S. Spatz, César Caraballo, Shiwani Mahajan, Valeria Raparelli, Erica C. Leifheit, Yuan Lu, Harlan M. Krumholz, John A. Spertus, Gail D’Onofrio, Louise Pilote, Judith H. Lichtman

**Affiliations:** 1 Department of Chronic Disease Epidemiology, Yale School of Public Health, New Haven, CT, United States of America; 2 Department of Emergency Medicine, Yale School of Medicine, New Haven, CT, United States of America; 3 Department of Biostatistics, Yale School of Public Health, New Haven, CT, United States of America; 4 Center for Methods in Implementation and Preventive Science, Yale University, New Haven, CT, United States of America; 5 Center for Outcomes Research and Evaluation, Yale School of Medicine, New Haven, CT, United States of America; 6 Section of Cardiovascular Medicine, Department of Internal Medicine, Yale School of Medicine, New Haven, CT, United States of America; 7 Department of Translational Medicine, University of Ferrara, Ferrara, Italy; 8 University Center for Studies on Gender Medicine, University of Ferrara, Ferrara, Italy; 9 Department of Health Policy and Management, Yale School of Public Health, New Haven, CT, United States of America; 10 Healthcare Institute for Innovations in Quality, University of Missouri–Kansas City, Kansas City, Missouri, United States of America; 11 Saint Luke’s Cardiovascular Outcomes Research, Saint Luke’s Mid America Heart Institute, Kansas City, Missouri, United States of America; 12 Center for Outcomes Research and Evaluation, Research Institute, McGill University Health Centre, Montreal, Quebec, Canada; 13 Department of Medicine and Research Institute of the McGill University Health Centre, Montreal, Quebec, Canada; Kurume University School of Medicine, JAPAN

## Abstract

**Introduction:**

Despite evidence supporting the benefits of marriage on cardiovascular health, the impact of marital/partner status on the long-term readmission of young acute myocardial infarction (AMI) survivors is less clear. We examined the association between marital/partner status and 1-year all-cause readmission and explored sex differences among young AMI survivors.

**Methods:**

Data were from the VIRGO study (Variation in Recovery: Role of Gender on Outcomes of Young AMI Patients), which enrolled young adults aged 18–55 years with AMI (2008–2012). The primary end point was all-cause readmission within 1 year of hospital discharge, obtained from medical records and patient interviews and adjudicated by a physician panel. We performed Cox proportional hazards models with sequential adjustment for demographic, socioeconomic, clinical, and psychosocial factors. Sex-marital/partner status interaction was also tested.

**Results:**

Of the 2,979 adults with AMI (2002 women [67.2%]; mean age 48 [interquartile range, 44–52] years), unpartnered individuals were more likely to experience all-cause readmissions compared with married/partnered individuals within the first year after hospital discharge (34.6% versus 27.2%, hazard ratio [HR] = 1.31; 95% confidence interval [CI], 1.15–1.49). The association attenuated but remained significant after adjustment for demographic and socioeconomic factors (adjusted HR, 1.16; 95% CI, 1.01–1.34), and it was not significant after further adjusting for clinical factors and psychosocial factors (adjusted HR, 1.10; 95%CI, 0.94–1.28). A sex-marital/partner status interaction was not significant (p = 0.69). Sensitivity analysis using data with multiple imputation and restricting outcomes to cardiac readmission yielded comparable results.

**Conclusions:**

In a cohort of young adults aged 18–55 years, unpartnered status was associated with 1.3-fold increased risk of all-cause readmission within 1 year of AMI discharge. Further adjustment for demographic, socioeconomic, clinical, and psychosocial factors attenuated the association, suggesting that these factors may explain disparities in readmission between married/partnered versus unpartnered young adults. Whereas young women experienced more readmission compared to similar-aged men, the association between marital/partner status and 1-year readmission did not vary by sex.

## Introduction

Despite an overall reduction in cardiovascular disease (CVD) prevalence and acute myocardial infarction (AMI) mortality [1], rates of AMI hospitalization in young adults (≤55 years) have increased over the last two decades [2]. Hospital readmission remains frequent across all age groups of AMI survivors, with an overall 24% readmission rate within 90 days post-AMI [3]. In a recent study of US young adults with AMI, about one-third had at least 1 hospitalization in the year after discharge, and young women experienced more adverse events than men [4]. The risk profile for readmission among younger and older patients may be different, as suggested by a study using data from the 2013 National Readmission Database, where the effect of sex was more prominent in the younger age group [3]. Socio-demographic and psychosocial characteristics have been suggested to play important roles in predicting the risk of 1-year readmission for younger adults with AMI [5], yet little is known about the impact of marital/partner status on their long-term risk of readmission.

Marriage has long been known to offer cardiovascular health benefits, including its association with lower risk of AMI incidence [6], in-hospital and long-term mortality [7–10], and recurrent events [11–13]. Committed relationships that are not based on formal legal unions, such as domestic partners and common-law marriages, may also convey benefits but are less commonly described in prior studies [7, 9, 10, 12]. Moreover, prior research has largely focused on older populations, been conducted in foreign countries, and not explored readmission beyond the first month of discharge [11, 12]. There is a paucity of data on the impact of marital/partner status on the long-term readmission outcomes of younger AMI patients. In addition, although evidence suggests that women may not benefit from marriage to the same extent as men regarding mortality outcomes [14, 15], less is known about whether there are sex differences in the degree of “protection” conferred by marriage/partnership in a younger population with AMI and as assessed by hospital readmission.

To address this gap in knowledge, we examined the association between marital/partner status and all-cause and cardiac readmission within 1 year of hospital discharge among a cohort of AMI survivors 18–55 years of age in the United States. A secondary aim was to explore potential subgroup differences in the association by sex.

## Materials and methods

### Study population

We used data from the VIRGO study (Variation in Recovery: Role of Gender on Outcomes of Young AMI Patients), the largest prospective, multicenter cohort study designed to understand factors associated with adverse outcomes in younger adults (≤55 years) with AMI [16]. Between August 21, 2008 and May 1, 2012, a total of 2,979 participants were recruited from 103 US hospitals using a 2:1 female-to-male enrollment design. The methodology of VIRGO has been described elsewhere [16]. In brief, eligible participants had elevated cardiac enzymes (troponin or creatine kinase, with at least one of these biomarkers >99^th^ percentile of the upper reference limit) at the recruiting center within 24 hours of admission, and presented with other evidence supporting the diagnosis of AMI, including either symptoms of ischemia or electrocardiogram changes indicative of new ischemia (new ST-T changes, new or presumably new left bundle branch block, or the development of pathological Q waves). Patients were excluded if their elevated cardiac markers were due to a complication of elective coronary revascularization or their AMI was caused by physical trauma. Individuals were ineligible if they were incarcerated, did not speak English or Spanish, or were unable to provide informed consent or be contacted for follow-up.

This study followed the Strengthening the Reporting of Observational Studies in Epidemiology (STROBE) reporting guideline (**S1 Table**). De-identified data were used for the current study. All supporting data of this study are available upon reasonable request from Dr Yuan Lu (y.lu@yale.edu). Institutional review board approval was obtained from the Yale Human Investigation Committee as well as each participating institution, and individuals provided written informed consent for their study participation.

### Assessment of marital/partner status and other covariates

Baseline data were collected by medical chart abstraction and standardized in-person interviews administered by trained personnel during the index AMI admission. Marital/Partner status was collected during the patient enrollment interview through a question of “Which best describes your current marital status” and was categorized into “married/partnered” (having a response of “married” or “living as married/living with partner”) or “unpartnered” (having a response of “divorced”, “separated”, “widowed”, or “single”). In a secondary analysis, “unpartnered” status was further classified into “divorced/separated”, “widowed”, or “single”.

Demographic factors included sex (male/female), age (year, continuous), and self-reported race (non-Hispanic white/non-Hispanic black/Hispanic/other [ie, American Indian or Alaska Native and Asian or Pacific Islander]). Socioeconomic factors included education level, financial strain, employment status, and health insurance. Education level was categorized into less than high school, some high school, and more than high school. Financial strain was defined as having “just enough to make ends meet” or “not enough to make ends meet” (versus having some money left over) based on an individual’s response to the question “In general, how do your finances usually work out at the end of the month”.

Clinical characteristics considered in our study included cardiac risk factors (hypertension, dyslipidemia, diabetes, obesity, current smoker, and alcohol abuse), medical history (prior CVD [AMI, percutaneous coronary intervention, coronary artery bypass grafting, angina, heart failure, stroke, transient ischemic attack, or peripheral artery disease], renal dysfunction, and history of chronic obstructive pulmonary disease [COPD]), and disease severity (type of AMI [ST-elevation myocardial infarction/non-ST-elevation myocardial infarction], ejection fraction <40%, and hospital length of stay).

Psychosocial factors, including depression, low social support, and high stress burden, were assessed at baseline by validated measures or questionnaires. Depressive symptoms were measured using the 9-item version of the Patient Health Questionnaire [17], with an overall score of 10 or more indicating depression. Social support was measured using the 5-item Enhancing Recovery in Coronary Heart Disease Social Support Inventory [18]. Low social support was defined as a score of 3 or less on at least 2 Social Support Instrument items and a total score of 18 or less [19]. High stress burden was captured by answering “Fairly often” or “Very often” to the 14-item Perceived Stress Scale [20] question “In the last month, how often have you felt nervous and stressed?”

### Collection and adjudication of hospital readmission

The primary end point of this study was all-cause readmission within 1 year of hospital discharge. During the 1-year follow-up period, hospital readmissions were identified by the research coordinators at each site from medical record and self-report. The VIRGO adjudication process was supported through the use of a custom-developed Research Electronic Data Capture external module [21]. Adjudications of all-cause and cardiac readmission were completed by 5 physicians and an advanced practice registered nurse at Yale University who received extensive training and clear guidelines. The detailed process has been described elsewhere [5]. In sensitivity analysis, we restricted outcomes to cardiac readmission and results remained consistent.

### Statistical analysis

Baseline characteristics were compared between married/partnered versus unpartnered participants, overall and by sex, using *χ*^2^ test for dichotomous variables and Wilcoxon’s rank-sum test for continuous variables that did not follow a normal distribution. All variables had minimal missing values (<5%) except for financial strain (11%). In sensitivity analysis, multiple imputation by chained equations was applied to generate 10 imputed datasets on which estimates were calculated and pooled by the Rubin’s rule [22]. Modeling was performed both with and without the imputation of missing values, and because results were almost identical, we reported the complete case analysis in the main paper and presented the results from multiple imputation in **S2 Table**.

Time to readmission was compared between married/partnered and unpartnered groups using the log-rank test. To examine the independent association of marital/partner status with all-cause readmission over the subsequent 1 year after AMI, multivariable Cox proportional hazards models sequentially adjusted for 4 domains of covariates including demographics (age, sex, race), socioeconomic factors (education level, financial strain, employment status, and health insurance), clinical characteristics (cardiovascular risk factors, medical history, and disease severity), and psychosocial factors (depression, low social support, high stress burden). Covariates adjusted in the multivariable models were selected using a combination of clinical judgement and insights from the literature [5, 23, 24], with a detailed variable selection procedure described elsewhere [5]. Two-way interaction between marital/partner status and sex was also tested in the fully adjusted model using the Wald *χ*^2^ test. The proportional hazards assumption was checked by Schoenfeld residual with a global test p>0.05 indicating no violation.

Due to competing risk from non-cardiac readmissions in the sensitivity analysis, the Fine-Gray competing risk model was applied to examine the association between marital/partner status and the cumulative incidence of cardiac readmission within 1 year post-discharge. The same set of covariates considered in the Cox model were adjusted for in the Fine-Gray model.

All statistical analyses were conducted using R (Version 1.4.1106), with 2-tailed tests for statistical significance indicated by p = 0.05. Data analysis was performed from February to July 2022.

## Results

Among the 2,979 participants 18–55 years of age (median age 48 years, interquartile range 44–52), 42.8% were unpartnered (781 divorced/separated, 91 widowed, 423 single; 47% for women and 37.2% for men). Baseline characteristics are presented in **Table 1**.

**Table 1:** Baseline characteristics of the study participants by marital/partner status and sex.

	Married/Partnered (N = 1675)			Unpartnered (N = 1299)			P-value (unpartnered vs. married/partnered)
	Men (n = 610)	Women (n = 1065)	p-value	Men (n = 362)	Women (n = 937)	p-value	
***Demographics***							
**Age** (Median [q1, q3])	48 [44, 52]	48 [44, 52]	0.898	48 [43, 51]	48 [44, 52]	0.085	0.259
**Race**			0.319			<0.001	<0.001
Non-Hispanic white	476 (78.0%)	813 (76.3%)		252 (69.6%)	532 (56.8%)		
Non-Hispanic black	53 (8.7%)	134 (12.6%)		52 (14.4%)	275 (29.3%)		
Hispanic	43 (7.0%)	64 (6.0%)		39 (10.8%)	87 (9.3%)		
Other	38 (6.2%)	54 (5.1%)		19 (5.2%)	43 (4.6%)		
***Socioeconomic factors***							
**Education level**			0.148			0.005	0.007
Less than high school	10 (1.6%)	18 (1.7%)		6 (1.7%)	24 (2.6%)		
Some high school	220 (36.1%)	435 (40.8%)		183 (50.6%)	383 (40.9%)		
More than high school	376 (61.6%)	605 (56.8%)		169 (46.7%)	523 (55.8%)		
**Financial strain**			<0.001			0.029	<0.001
Yes	340 (55.7%)	686 (64.4%)		254 (70.2%)	729 (77.8%)		
No	212 (34.8%)	279 (26.2%)		55 (15.2%)	97 (10.4%)		
Missing	58 (9.5%)	100 (9.4%)		53 (14.6%)	111 (11.8%)		
**Employment status**			<0.001			0.075	<0.001
Unemployed	124 (20.3%)	434 (40.8%)		145 (40.1%)	440 (47.0%)		
Employed	483 (79.2%)	631 (59.2%)		217 (59.9%)	495 (52.8%)		
**Health insurance**			0.964			<0.001	<0.001
No	104 (17.0%)	187 (17.6%)		137 (37.8%)	246 (26.3%)		
Yes	504 (82.6%)	874 (82.1%)		221 (61.0%)	690 (73.6%)		
***Clinical factors (cardiac risk factors*, *medical history*, *and disease severity)***							
**Hypertension**	378 (62.0%)	659 (61.9%)	0.999	249 (68.8%)	687 (73.3%)	0.264	<0.001
**High cholesterol**	563 (92.3%)	889 (83.5%)	<0.001	340 (93.9%)	787 (84.0%)	<0.001	0.997
**Diabetes**	152 (24.9%)	379 (35.6%)	<0.001	107 (29.6%)	417 (44.5%)	<0.001	<0.001
**Obesity**	300 (49.2%)	545 (51.2%)	0.735	164 (45.3%)	562 (60.0%)	<0.001	0.013
**Physical inactivity**	163 (26.7%)	361 (33.9%)	0.009	132 (36.5%)	373 (39.8%)	0.575	<0.001
**Current smoker**	210 (34.4%)	338 (31.7%)	0.529	80 (22.1%)	263 (28.1%)	0.091	<0.001
**Alcohol abuse**	282 (46.2%)	287 (26.9%)	<0.001	173 (47.8%)	266 (28.4%)	<0.001	0.994
**Prior cardiovascular disease**	189 (31.0%)	348 (32.7%)	0.775	128 (35.4%)	367 (39.2%)	0.448	0.003
**Renal dysfunction**	41 (6.7%)	111 (10.4%)	0.040	42 (11.6%)	142 (15.2%)	0.254	<0.001
**COPD**	31 (5.1%)	130 (12.2%)	<0.001	31 (8.6%)	153 (16.3%)	0.002	<0.001
**Type of AMI**			<0.001			<0.001	0.417
STEMI	351 (57.5%)	501 (47.0%)		212 (58.6%)	417 (44.5%)		
NSTEMI	259 (42.5%)	564 (53.0%)		150 (41.4%)	520 (55.5%)		
**Ejection fraction<40%**			0.259			0.033	0.333
Yes	60 (9.8%)	132 (12.4%)		48 (13.3%)	79 (8.4%)		
No	535 (87.7%)	898 (84.3%)		304 (84.0%)	830 (88.6%)		
**Length of stay** (Median [q1, q3])	3 [2, 4]	3 [2, 4]	0.178	3 [2, 4]	3 [2, 5]	0.02	0.009
***Psychosocial factors***							
**Depression**	108 (17.7%)	362 (34.0%)	<0.001	103 (28.5%)	390 (41.6%)	<0.001	<0.001
**High stress burden**	211 (34.6%)	554 (52.0%)	<0.001	156 (43.1%)	531 (56.7%)	<0.001	<0.001
**Low social support**	82 (13.4%)	172 (16.2%)	0.341	130 (35.9%)	253 (27.0%)	0.007	<0.001
***1-year readmission outcomes***							
**All-cause readmission**	124 (20.3%)	331 (31.1%)	<0.001	97 (26.8%)	352 (37.6%)	0.001	<0.001
**Cardiac readmission**	94 (15.4%)	228 (21.4%)	0.001	78 (21.5%)	241 (25.7%)	0.293	0.002

**Fig 1:**
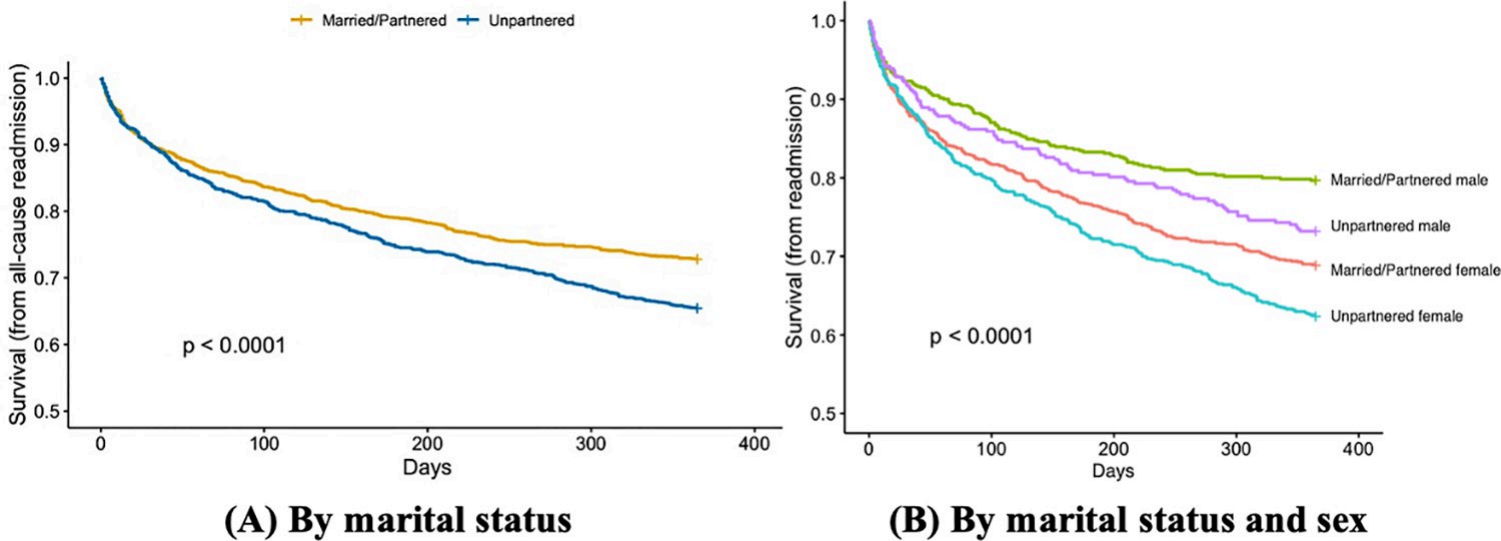
Kaplan-Meier curves for time to all-cause readmission. (A) By marital status and (B) By marital status and sex.

During the first year of follow-up, 904 patients had ≥1 all-cause readmission (50.3% were married), and 641 had ≥1 cardiac readmission (50.2% were married). Overall, compared to married/partnered individuals, those who were unpartnered had higher risk of all-cause readmission throughout the first year of recovery (**Fig 1A**). When further stratified by sex, married males had the lowest risk, while unmarried females had the highest (**Fig 1B**).

In multivariable analyses, compared with being married/partnered, being unpartnered was associated with a 24% higher risk of all-cause readmission during the first year of recovery after adjustment for demographic factors (age, sex and race) (aHR, 1.24; 95%CI, 1.08–1.42). The association attenuated after further adjusting for socioeconomic factors (education, financial strain, employment, insurance) (aHR, 1.16; 95%CI, 1.01–1.34), and further attenuated and became not statistically significant after adjusting for clinical factors (aHR, 1.11; 95%CI, 0.96–1.29) and psychosocial factors (aHR, 1.10; 95%CI, 0.94–1.28). Variables that were significant in the fully adjusted model included female sex, age, Hispanic race, financial strain, unemployment, diabetes, prior CVD, COPD, total length of stay, and depression. Details of the model output can be found in **Table 2**.

**Table 2:** Multivariable Cox regression models.

	Model 1 (R^2^ = 4.7%)	Model 2 (R^2^ = 9.1%)	Model 3 (R^2^ = 15%)	Model 4 (R^2^ = 16%)
**Marital status (Unpartnered vs. Married/Partnered)**	1.24 (1.08–1.42) *	1.16 (1.01–1.34) *	1.11 (0.96–1.29)	1.10 (0.94–1.28)
***Demographics***				
Female sex	1.52 (1.31–1.78) *	1.43 (1.21–1.69) *	1.36 (1.14–1.62) *	1.26 (1.05–1.51) *
Age, year	0.99 (0.98–1.00)	0.99 (0.98–1)	0.98 (0.97–0.99) *	0.98 (0.97–0.99) *
Race (ref: non-Hispanic white)	-	-	-	-
Non-Hispanic black	1.22 (1.04–1.44) *	1.18 (0.98–1.4)	1.15 (0.95–1.38)	1.19 (0.98–1.44)
Hispanic	0.66 (0.50–0.88) *	0.70 (0.52–0.95) *	0.64 (0.47–0.88) *	0.62 (0.45–0.87) *
Other race/ethnicity	0.84 (0.61–1.17)	0.89 (0.63–1.26)	0.87 (0.61–1.25)	0.91 (0.63–1.32)
***Socioeconomic factors***				
Education (ref: less than high school)		-	-	-
Some high school		0.91 (0.55–1.51)	1.04 (0.61–1.78)	0.97 (0.56–1.68)
More than high school		0.86 (0.52–1.44)	1.00 (0.58–1.70)	0.93 (0.53–1.61)
Financial strain		1.41 (1.15–1.72) *	1.32 (1.08–1.63) *	1.33 (1.07–1.64) *
Unemployment		1.49 (1.30–1.72) *	1.30 (1.11–1.54) *	1.30 (1.10–1.52) *
No health insurance		1.23 (1.04–1.47) *	1.12 (0.93–1.34)	1.14 (0.95–1.37)
***Clinical factors (cardiac risk factors*, *medical history*, *and disease severity)***				
Hypertension			1.05 (0.88–1.25)	1.01 (0.84–1.21)
High cholesterol			1.13 (0.89–1.42)	1.08 (0.85–1.37)
Diabetes			1.31 (1.12–1.54) *	1.33 (1.13–1.56) *
Obesity			0.89 (0.77–1.04)	0.88 (0.75–1.02)
Physical inactivity			1.08 (0.93–1.25)	1.05 (0.9–1.23)
Current smoking			1.07 (0.91–1.27)	1.11 (0.93–1.31)
Alcohol abuse			0.96 (0.82–1.13)	0.98 (0.83–1.16)
Prior cardiovascular disease			1.23 (1.06–1.44) *	1.22 (1.03–1.43) *
Renal dysfunction			1.19 (0.96–1.47)	1.23 (0.99–1.52)
COPD			1.35 (1.11–1.65) *	1.32 (1.07–1.62) *
STEMI			1.00 (0.87–1.16)	1.04 (0.89–1.21)
Ejection fraction <40%			0.92 (0.73–1.16)	0.89 (0.7–1.13)
Total length of stay			1.03 (1.02–1.05) *	1.03 (1.01–1.05) *
***Psychosocial factors***				
Depression				1.35 (1.13–1.61) *
Low social support				0.97 (0.81–1.16)
High stress burden				1.07 (0.91–1.26)

*p<0.05 indicating statistical significance.

Note: Data are presented as hazard ratio (95% confidence interval). Model 1 adjusted for demographics. Model 2 adjusted for demographic and socioeconomic factors. Model 3 adjusted for demographic, socioeconomic, and clinical factors. Model 4 adjusted for demographic, socioeconomic, clinical, and psychosocial factors. Covariates were pre-selected based on prior literature and clinical implications. Interaction between marital/partner status and sex was tested and was not significant in the fully adjusted models (p = 0.628). The fully adjusted model did not violate the proportional hazards assumption (global p>0.05).

In the fully adjusted model, the 2-way interaction between marital/partner status and sex was not significant (p = 0.628). To provide additional information on the direction of the interaction, the fully adjusted models were also stratified by sex. Details of the sex-specific models can be found in **S3 Table**.

Sensitivity analysis using data with multiple imputation and restricting outcomes to cardiac readmission yielded comparable results (**S2 and S4 Tables**). In the fully adjusted model with imputed data, no health insurance was also associated with 1-year all-cause readmission. When restricting outcomes to cardiac readmission using the Fine-Gray model, financial strain and depression were no longer significant in the fully adjusted model.

In a secondary analysis further classifying unpartnered participants into divorced/separated, widowed, and single subgroups, a similar pattern was found for divorced/separated individuals and the overall unpartnered group when compared with married/partnered participants (**Table 3**). Widowed individuals had the highest risk of 1-year all-cause readmission when compared to married/partnered individuals, yet the association was not statistically significant due to the small number of widowed participants in the current study. No association was found among single individuals.

**Table 3:** Association between marital/partner status subgroup and 1-year all-cause readmission.

	Model 1	Model 2	Model 3	Model 4
Married/Partnered (1675)	ref	ref	ref	Ref
**Unpartnered (1299)**	1.24 (1.08–1.42) *	1.16 (1.01–1.34) *	1.11 (0.96–1.29)	1.10 (0.94–1.28)
**Divorced/Separated (781)**	1.26 (1.08–1.46) *	1.20 (1.02–1.41) *	1.17 (0.98–1.38)	1.15 (0.96–1.37)
**Widowed (91)**	1.39 (0.99–1.95)	1.29 (0.91–1.84)	1.09 (0.76–1.57)	1.04 (0.72–1.51)
**Single (423)**	1.17 (0.96–1.43)	1.06 (0.85–1.31)	1.00 (0.80–1.26)	1.00 (0.80–1.28)

*p<0.05 indicating statistical significance.

Note: Data are presented as hazard ratio (95% confidence interval). Model 1 adjusted for demographics. Model 2 adjusted for demographic and socioeconomic factors. Model 3 adjusted for demographic, socioeconomic, and clinical factors. Model 4 adjusted for demographic, socioeconomic, clinical, and psychosocial factors. Covariates were pre-selected based on prior literature and clinical implications.

## Discussion

In a cohort of young adults with AMI in the United States, unpartnered individuals had a 1.3-fold higher 1-year readmission rate compared to married/partnered individuals. The association attenuated and remained significant after adjusting for demographic and socioeconomic factors, but it was not significant after further adjusting for clinical and psychosocial factors. A sex difference was not evident in the fully adjusted model.

Prior research has generally supported improved survival and fewer recurrent events in married individuals compared to their unmarried counterparts within 1 year post-AMI [7–13]. However, only a few studies investigated the impact of marital/partner status on post-event health outcomes beyond mortality among AMI survivors, with younger patients being underrepresented [11–13]. Our study addresses this important knowledge gap using adjudicated readmission data from a large nationwide cohort of young adults with AMI in the United States. Compared to an Israeli study of AMI patients with a mean age of 64 years [11], which found no unadjusted association between marital status and 30-day readmission (17.8% among married, 19.1% among nonmarried, p = 0.28), our study showed a higher unadjusted all-cause readmission rate within 1 year after AMI among unpartnered individuals compared to those who were married/partnered. Differences in the results could be due to a longer follow up time in our study and different participant characteristics (eg, younger age, different culture).

Previous studies that reported an independent association between marital status and AMI outcomes have not generally accounted for socioeconomic or psychosocial factors in their analyses [11–13]. However, mounting evidence has demonstrated that these factors are more powerful predictors of adverse outcomes following AMI than physical health indicators, especially in a younger population [5, 25]. Our finding of the association being attenuated after adjusting for socioeconomic status and clinical factors supports the roles of both clinical (ie, diabetes, CVD history, COPD, length of stay) and socioeconomic components (ie, financial and employment status) in explaining the complicated relationship between marriage/partnership and health outcomes. Further, these findings align with prevailing theories that explain the mechanism of marriage protection. First, the marriage selection theory suggests that healthier people may be more likely to get and stay married [26]. In our study, we also observed poorer health at baseline, including clinical, behavioral, and psychosocial factors, among unpartnered compared to married/partnered individuals. Second, the social causation theory centers on the health benefits from spousal support with regard to treatment adherence, lifestyle changes, and greater socioeconomic resources, which make healthy behaviors affordable [27, 28]. In our study, while further adjusting for psychosocial factors did not substantially change the results, findings provided a more comprehensive risk factor profile for young adults with AMI, where unpartnered individuals were more likely to have depression, low social support, and high stress burden at baseline. While aspects of marriage such as marital quality were beyond the scope of the current study, research using VIRGO data demonstrated that stress experienced in a marriage or partnered relationship was associated with worse 1-year recovery for young AMI survivors [25]. Our results lend support to the marriage selection and social causation theories in a younger population with AMI, but they should be interpreted with caution since our exploratory analysis can only generate associational evidence. Future research is encouraged to include a formal mediation analysis to understand the complex relationship and potential causal pathway associated with these findings.

Studies on sex differences in the impact of marital/partner status on cardiovascular outcomes have yielded mixed findings, with the majority of prior work supporting a greater marital benefit for men than women [6, 10, 13, 15]. On the contrary, our study did not find such a sex difference in the association in a younger population. The potential mechanism for such findings is likely to be the offset of biological and psychosocial effects. Physiologically, women were protected against heart disease by sex hormones such as estrogen that reduces circulatory levels of harmful cholesterol vs testosterone that increases the concentrations of low-density lipoprotein and inflammatory markers that affect atherosclerosis and stroke progression [29]. From a psychosocial perspective, however, women had distinct vulnerabilities, including unique sources of psychosocial stress and discrimination, increased perceived stress during adulthood, and 2-fold greater lifetime prevalence of depression and anxiety disorders compared with men [30–32]. This was also evident in the current study where unmarried women had the highest likelihood to have depression and high stress burden. Taken together, while our study found the impact of marital/partner status was equal for younger men and women, it might involve a mixture of biological and psychosocial effects that warrant further investigation.

### Study implications

This study adds to the understanding of the association between marital/partner status and readmission outcomes up to 1 year after AMI in younger adults. Readmission presents a complex interaction between patients, community, environment, and healthcare system, and it is an important measure indicating health outcomes and disease burden. Usually perceived as a demographic variable, marital/partner status adds an important dimension of social support and is also an easily attainable indicator. Clinicians may consider incorporating patients’ marital/partner status along with other socio-demographic factors into risk assessment and decision-making to create a more patient-centered practice. Our study may also inform potential interventions based on the social and psychological context of younger adults with AMI. For example, support groups or secondary prevention programs could widen participation of unpartnered individuals to improve their psychosocial well-being and recovery.

### Limitations

Limitations of this study merit discussion. Self-reported readmission was validated with retrospective chart review, but misclassification bias may still be present. Although our study included an extensive array of demographic, socioeconomic, and clinical factors, residual confounding due to unmeasured characteristics that differ by marital/partner status may still bias the results. Our modeling approach generated only associational evidence instead of causation, and therefore, findings should be interpreted with caution. Participants enrolled in the VIRGO study may not reflect those who did not enroll in the study or were hospitalized at other institutions.

## Conclusions

Unpartnered individuals with AMI had a 1.3 times higher risk of all-cause readmission within 1 year after hospital discharge compared to their married/partnered counterparts. This association attenuated yet remained significant after adjusting for demographic and socioeconomic factors. However, after further adjustment for clinical and psychosocial factors, the association was no longer significant. There was no difference by sex in the relationship between married/partnered status and 1-year readmission. Further study is needed to explore the potential causal relationships underlying these findings.

## Supporting information

S1 Table STROBE statement checklist. (DOCX)

S2 Table Multivariable Cox regression models using multiple imputed data. (DOCX)

S3 Table Sex-specific Cox regression models. (DOCX)

S4 Table Multivariable fine-gray models (cardiac readmission). (DOCX)
