# Supplementary material for: Association of marital/partner status with hospital readmission among young adults with acute myocardial infarction
Source: PLoS One. 2024 Jan 26;19(1):e0287949. doi: 10.1371/journal.pone.0287949 (PMC10817183; doi:10.1371/journal.pone.0287949)
Supplement: S2 Table — (DOCX) [file pone.0287949.s002.docx]

**S2 Table. Multivariable Cox regression models using multiple imputed data**

|  | **Model 1** | **Model 2** | **Model 3** | **Model 4** |
| --- | --- | --- | --- | --- |
| **Marital status (Unpartnered vs. Married/Partnered)** | 1.24 (1.08, 1.42) * | 1.17 (1.02, 1.35) * | 1.12 (0.97, 1.28) | 1.1 (0.96-1.26) |
| ***Demographics*** | | | |  |
| Female sex | 1.52 (1.31-1.78) * | 1.39 (1.19, 1.63) * | 1.33 (1.13, 1.56) * | 1.26 (1.07-1.48) * |
| Age (year) | 0.99 (0.98-1.00) | 0.99 (0.98, 1.00) | 0.99 (0.98, 1.00)* | 0.99 (0.98-1.00) * |
| Race (ref: non-Hispanic white) | - | - | - | - |
| Non-Hispanic black | 1.22 (1.04-1.44) * | 1.14 (0.97, 1.35) | 1.09 (0.92, 1.30) | 1.15 (0.97-1.37) |
| Hispanic | 0.67 (0.51-0.89) * | 0.66 (0.50, 0.89) * | 0.63 (0.47, 0.84) * | 0.63 (0.47-0.85) * |
| Other race/ethnicity | 0.85 (0.61-1.17) | 0.86 (0.62, 1.19) | 0.79 (0.57, 1.10) | 0.84 (0.6-1.16) |
| ***Socioeconomic factors*** | | | |  |
| Education (ref: less than high school) |  | - | - | - |
| Some high school |  | 1.10 (0.66, 1.83) | 1.24 (0.74, 2.08) | 1.24 (0.74-2.07) |
| More than high school |  | 1.07 (0.64, 1.79) | 1.24 (0.74, 2.08) | 1.23 (0.73-2.06) |
| Financial strain |  | 1.39 (1.15, 1.67) * | 1.31 (1.08, 1.59) * | 1.29 (1.04-1.60) * |
| Unemployment |  | 1.50 (1.30, 1.72) * | 1.29 (1.11, 1.49) * | 1.23 (1.06-1.43) * |
| No health insurance |  | 1.26 (1.07, 1.48) * | 1.16 (0.99, 1.37) | 1.19 (1.01-1.41) * |
| ***Clinical factors (cardiac risk factors, medical history, and disease severity)*** | | | |  |
| Hypertension |  |  | 1.08 (0.92, 1.27) | 1.05 (0.89-1.23) |
| High cholesterol |  |  | 1.12 (0.91, 1.39) | 1.1 (0.89-1.37) |
| Diabetes |  |  | 1.24 (1.07, 1.44) * | 1.23 (1.06-1.43) * |
| Obesity |  |  | 0.93 (0.81, 1.06) | 0.93 (0.81-1.07) |
| Physical inactivity |  |  | 1.11 (0.97, 1.28) | 1.07 (0.93-1.23) |
| Current smoking |  |  | 1.03 (0.88, 1.20) | 1.06 (0.91-1.24) |
| Alcohol abuse |  |  | 0.96 (0.83, 1.12) | 0.97 (0.83-1.12) |
| Prior cardiovascular disease |  |  | 1.22 (1.06, 1.41) * | 1.21 (1.05-1.4) * |
| Renal dysfunction |  |  | 1.15 (0.95, 1.40) | 1.16 (0.96-1.41) |
| COPD |  |  | 1.28 (1.06, 1.54) * | 1.21 (1.00-1.46) * |
| STEMI |  |  | 0.96 (0.84, 1.10) | 0.98 (0.86-1.12) |
| Ejection fraction <40% |  |  | 0.99 (0.81, 1.22) | 0.98 (0.80-1.21) |
| Total length of stay |  |  | 1.03 (1.02, 1.04) * | 1.03 (1.02-1.04) * |
| **Psychosocial factors** | | | | |
| Depression |  |  |  | 1.35 (1.15-1.57) * |
| Low social support |  |  |  | 0.98 (0.83-1.15) |
| High stress burden |  |  |  | 1.11 (0.95-1.28) |

*p<0.05 indicating statistical significance.

Note: Data are presented as hazard ratio (95% confidence interval). Model 1 adjusted for demographics. Model 2 adjusted for demographic and socioeconomic factors. Model 3 adjusted for demographic, socioeconomic, and clinical factors. Model 4 adjusted for demographic, socioeconomic, clinical, and psychosocial factors. Covariates were pre-selected based on prior literature and clinical implications. Interaction between marital/partner status and sex was tested and was not significant in the fully adjusted models (p=0.69). The fully adjusted model did not violate the proportional hazards assumption (global p>0.05).
