# Supplementary material for: Association of marital/partner status with hospital readmission among young adults with acute myocardial infarction
Source: PLoS One. 2024 Jan 26;19(1):e0287949. doi: 10.1371/journal.pone.0287949 (PMC10817183; doi:10.1371/journal.pone.0287949)
Supplement: S3 Table — (DOCX) [file pone.0287949.s003.docx]

**S3 Table. Sex-specific Cox regression models**

| **Variables** | **HR (95%CI)** |  |
| --- | --- | --- |
|  | **Female only** | **Male only** |
| **Marital status (Unpartnered vs. Married/Partnered)** | 1.08 (0.77-1.49) | 1.07 (0.77-1.49) |
| ***Demographics*** | | |
| Age | 0.98 (0.96-1.01) | 0.99 (0.96-1.02) |
| Race (non-Hispanic black vs. non-Hispanic white) | 0.94 (0.57-1.54) | 0.94 (0.57-1.54) |
| Race (Hispanic vs. non-Hispanic white) | 0.76 (0.54-1.09) | 0.29 (0.12-0.67) * |
| Race (other vs. non-Hispanic white) | 0.88 (0.57-1.36) | 0.86 (0.41-1.67) |
| ***Socioeconomic factors*** | | |
| Education (some high school vs. less than high school) | 0.86 (0.49-1.52) | 3.03 (0.41-22.35) |
| Education (more than high school vs. less than high school) | 0.83 (0.47-1.46) | 3.04 (0.41-22.42) |
| Financial strain | 1.16 (0.91-1.48) | 1.72 (1.14-2.59) * |
| Unemployed | 1.30 (1.09-1.54) * | 1.32 (0.94-1.85) |
| Uninsured | 1.15 (0.93-1.41) | 0.99 (0.69-1.42) |
| ***Clinical factors (cardiac risk factors, medical history, and disease severity)*** | | |
| Hypertension | 1.05 (0.86-1.29) | 1.04 (0.73-1.48) |
| High cholesterol | 1.17 (0.90-1.51) | 1.00 (0.55-1.81) |
| Diabetes | 1.34 (1.12-1.61) * | 1.22 (0.87-1.73) |
| Obesity | 0.96 (0.80-1.14) | 0.71 (0.52-0.98) * |
| Physical inactivity | 1.09 (0.92-1.30) | 1.06 (0.76-1.47) |
| Current smoking | 1.10 (0.91-1.33) | 0.95 (0.66-1.36) |
| Alcohol abuse | 0.91 (0.75-1.10) | 1.08 (0.80-1.47) |
| Prior cardiovascular disease | 1.21 (1.01-1.45) * | 1.33 (0.96-1.84) |
| Renal dysfunction | 1.26 (1.00-1.59) | 1.00 (0.6-1.67) |
| COPD | 1.38 (1.11-1.72) * | 1.28 (0.76-2.17) |
| STEMI | 1.06 (0.90-1.26) | 0.86 (0.64-1.16) |
| Ejection fraction<40% | 0.84 (0.64-1.10) | 1.11 (0.70-1.75) |
| Total length of stay | 1.03 (1.01-1.05) * | 1.04 (1.01-1.08) * |
| ***Psychosocial factors*** |  |  |
| Depression | 1.46 (1.20-1.78) * | 1.11 (0.75-1.64) |
| Low social support | 0.89 (0.72-1.10) | 1.20 (0.83-1.72) |
| High stress burden | 1.11 (0.92-1.35) | 0.95 (0.68-1.33) |

*p<0.05 indicating statistical significance.

Note: Sex-specific models adjusted for demographic, socioeconomic, clinical, and psychosocial factors. Covariates were pre-selected based on prior literature and clinical implications. Both fully adjusted models did not violate the proportional hazards assumption (global p>0.05).
