# Supplementary material for: Association of marital/partner status with hospital readmission among young adults with acute myocardial infarction
Source: PLoS One. 2024 Jan 26;19(1):e0287949. doi: 10.1371/journal.pone.0287949 (PMC10817183; doi:10.1371/journal.pone.0287949)
Supplement: S4 Table — (DOCX) [file pone.0287949.s004.docx]

**S4 Table. Multivariable Fine-Gray models (cardiac readmission)**

|  | **Model 1** | **Model 2** | **Model 3** | **Model 4** |
| --- | --- | --- | --- | --- |
| **Marital status (Unpartnered vs. Married/Partnered)** | 1.26 (1.08, 1.48) * | 1.17 (0.99, 1.39) | 1.12 (0.94, 1.34) | 1.1 (0.91-1.32) |
| ***Demographics*** | | | |  |
| Female sex | 1.33 (1.12-1.59) * | 1.29 (1.07, 1.57) * | 1.24 (1.01, 1.53) * | 1.19 (0.97-1.41) * |
| Age (year) | 0.99 (0.98-1.00) | 0.99 (0.97, 1.00) | 0.98 (0.96, 0.99) * | 0.98 (0.96-0.99) * |
| Race (ref: non-Hispanic white) | - | - | - | - |
| Non-Hispanic black | 1.22 (1.04-1.44) * | 1.14 (0.97, 1.35) | 1.03 (0.93, 1.15) | 1.02 (0.91-1.14) |
| Hispanic | 0.67 (0.51-0.89) * | 0.66 (0.50, 0.89) * | 0.63 (0.47, 0.84) * | 0.62 (0.45-0.83) * |
| Other race/ethnicity | 0.85 (0.61-1.17) | 0.86 (0.62, 1.19) | 0.79 (0.57, 1.10) | 0.80 (0.55-1.12) |
| ***Socioeconomic factors*** | | | |  |
| Education (ref: less than high school) |  | - | - | - |
| Some high school |  | 0.96 (0.82, 1.12) | 1.00 (0.85, 1.18) | 0.99 (0.84-1.17) |
| More than high school |  | 1.07 (0.64, 1.79) | 1.24 (0.74, 2.08) | 1.23 (0.72-2.06) |
| Financial strain |  | 1.33 (1.05, 1.69) * | 1.23 (0.96, 1.57) | 1.25 (0.96-1.61) |
| Unemployment |  | 1.56 (1.30, 1.81) * | 1.33 (1.10, 1.61) * | 1.31 (1.07-1.59) * |
| No health insurance |  | 1.13 (0.92, 1.39) | 1.05 (0.85, 1.29) | 1.06 (0.85-1.32) |
| ***Clinical factors (cardiac risk factors, medical history, and disease severity)*** | | | |  |
| Hypertension |  |  | 1.23 (0.99, 1.53) | 1.20 (0.96-1.51) |
| High cholesterol |  |  | 1.22 (0.91, 1.63) | 1.17 (0.87-1.57) |
| Diabetes |  |  | 1.25 (1.04, 1.51) * | 1.27 (1.05-1.53) * |
| Obesity |  |  | 0.87 (0.72, 1.04) | 0.84 (0.70-1.02) |
| Physical inactivity |  |  | 1.05 (0.88, 1.26) | 1.05 (0.87-1.27) |
| Current smoking |  |  | 0.96 (0.79, 1.17) | 1.00 (0.81-1.23) |
| Alcohol abuse |  |  | 0.98 (0.82, 1.19) | 1.00 (0.83-1.21) |
| Prior cardiovascular disease |  |  | 1.27 (1.05, 1.53) * | 1.26 (1.04-1.53) * |
| Renal dysfunction |  |  | 1.20 (0.94, 1.54) | 1.21 (0.94-1.57) |
| COPD |  |  | 1.36 (1.08, 1.73) * | 1.33 (1.04-1.71) * |
| STEMI |  |  | 1.00 (0.88, 1.47) | 1.03 (0.86-1.24) |
| Ejection fraction <40% |  |  | 0.99 (0.81, 1.22) | 1.08 (0.82-1.42) |
| Total length of stay |  |  | 1.02 (1.00, 1.04) | 1.01 (0.99-1.03) |
| **Psychosocial factors** |  |  |  |  |
| Depression |  |  |  | 1.17 (0.95-1.45) |
| Low social support |  |  |  | 1.02 (0.82-1.27) |
| High stress burden |  |  |  | 1.06 (0.88-1.29) |

*p<0.05 indicating statistical significance.

Note: Data are presented as hazard ratio (95% confidence interval). Model 1 adjusted for demographics. Model 2 adjusted for demographic and socioeconomic factors. Model 3 adjusted for demographic, socioeconomic, and clinical factors. Model 4 adjusted for demographic, socioeconomic, clinical, and psychosocial factors. Covariates were pre-selected based on prior literature and clinical implications. Interaction between marital/partner status and sex was tested and was not significant in the fully adjusted models (p=0.729).
